# Performance of methods for meta-analysis of diagnostic test accuracy with few studies or sparse data

**DOI:** 10.1177/0962280215592269

**Published:** 2015-06-26

**Authors:** Yemisi Takwoingi, Boliang Guo, Richard D Riley, Jonathan J Deeks

**Affiliations:** 1 Public Health, Epidemiology and Biostatistics, University of Birmingham, Edgbaston, Birmingham, UK; 2 School of Medicine, University of Nottingham, Nottingham, UK; 3 Research Institute of Primary Care and Health Sciences, Keele University, Staffordshire, UK

**Keywords:** Diagnostic accuracy, meta-analysis, hierarchical models, HSROC model, bivariate model, sensitivity, specificity, diagnostic odds ratio, sparse data, random effects

## Abstract

Hierarchical models such as the bivariate and hierarchical summary receiver operating characteristic (HSROC) models are recommended for meta-analysis of test accuracy studies. These models are challenging to fit when there are few studies and/or sparse data (for example zero cells in contingency tables due to studies reporting 100% sensitivity or specificity); the models may not converge, or give unreliable parameter estimates. Using simulation, we investigated the performance of seven hierarchical models incorporating increasing simplifications in scenarios designed to replicate realistic situations for meta-analysis of test accuracy studies. Performance of the models was assessed in terms of estimability (percentage of meta-analyses that successfully converged and percentage where the between study correlation was estimable), bias, mean square error and coverage of the 95% confidence intervals. Our results indicate that simpler hierarchical models are valid in situations with few studies or sparse data. For synthesis of sensitivity and specificity, univariate random effects logistic regression models are appropriate when a bivariate model cannot be fitted. Alternatively, an HSROC model that assumes a symmetric SROC curve (by excluding the shape parameter) can be used if the HSROC model is the chosen meta-analytic approach. In the absence of heterogeneity, fixed effect equivalent of the models can be applied.

## 1 Introduction

Meta-analysis of test accuracy studies aims to produce reliable evidence about the diagnostic accuracy of a medical test from multiple studies addressing the same question. The bivariate model^1^ and the hierarchical summary receiver operating characteristic (HSROC) model^2^ are the two approaches recommended for meta-analysis when a sensitivity and specificity pair is available for each study.^3–5^ These hierarchical models possess theoretical advantages over simpler methods for meta-analysis of test accuracy studies but fitting them is not trivial. The models are often fitted using a frequentist approach that relies on likelihood based methods for the estimation of five parameters. Solving the likelihood equations requires an iterative process and in certain circumstances, for instance when there are few studies and/or sparse data (e.g. zero cells due to perfect sensitivity and/or specificity) in a meta-analysis, the models fail to converge or they converge but give unreliable parameter estimates with one or more missing standard errors. These issues are often encountered by meta-analysts^6^ and there is uncertainty about how to proceed with meta-analysis in such situations.

Academic illustrations of the application of hierarchical methods have typically involved large meta-analyses.^1,2,4,7–13^ In contrast, our experience of supporting Cochrane and non-Cochrane diagnostic test accuracy review authors suggest that small meta-analyses or sparse data often occur and pose a challenge to these data hungry hierarchical models. Others have also noted the problem of non-convergence.^8,10,14–16^ Despite the increasing uptake of these models, a recent survey has suggested a lack of clarity about recommended methods for meta-analysis and a need for guidance.^16^ In this paper, using simulation, we evaluate the performance of hierarchical models for meta-analysis of diagnostic accuracy studies, and we develop recommendations for their use. Because sensitivity and specificity are the test accuracy measures most commonly used in meta-analyses,^17^ we consider only methods for synthesis of these measures. Other measures such as likelihood ratios can be derived from functions of the bivariate or HSROC model parameters.

The outline of this paper is as follows. In section 2 we briefly describe common methods used for meta-analysis when each study contributes a single 2 x 2 table of the results of an index test cross classified with a reference standard. In section 3 we outline two motivating examples where the bivariate model failed to converge, and we apply simpler forms of the hierarchical models to resolve this. In section 4 we describe the simulation study and present the results for full and simplified hierarchical models. In section 5 we discuss our findings and conclude with recommendations for selecting an appropriate meta-analytic approach in practice.

## 2 Methods for meta-analysis of diagnostic accuracy studies

### 2.1 Univariate pooling methods

Univariate fixed effect or random effects meta-analytic methods pool sensitivity and specificity separately, ignoring any correlation that may exist between the two measures. Fixed effect models assume homogeneity while random effects models assume variability in test accuracy beyond sampling error alone by allowing each study to have its own test accuracy, i.e. the model includes a between study variance component ($\sigma^{2}$). Let $\mu_{\mathrm{Ai}}$ and $\mu_{\mathrm{Bi}}$ be the logit sensitivity and logit specificity, and $\sigma_{\mathrm{Ai}}^{2}$ and $\sigma_{\mathrm{Bi}}^{2}$ their variances for the *i*th study (*i* = 1, 2, … , *N*), then the models for sensitivity and specificity are specified as

(1) $$\mu_{\mathrm{Ai}}∼N(\mu_{A},\sigma_{A}^{2}),\mu_{\mathrm{Bi}}∼N(\mu_{B},\sigma_{B}^{2})$$

The simplest and most commonly used random effects method is the DerSimonian and Laird approach which uses a normal distribution to model within study variability. Logit transformed sensitivity or specificity and the within study variance are undefined when there are zero cells. A continuity correction (typically 0.5) is applied, leading to a downward bias in test accuracy.^6^ Therefore, univariate methods that use a binomial distribution to model within study variability are preferred. However, these logistic models are seldom used in practice probably due to lack of awareness of the methods or software limitations.

### 2.2 Summary receiver operating characteristic regression

The summary receiver operating characteristic (SROC) curve approach developed by Moses et al.^18^ accounts for possible heterogeneity in threshold. It uses a logistic transformation of the true positive and false positive rates (TPR and FPR) and linear regression to model the relationship between test accuracy and the proportion test positive (related to threshold). If accuracy does not depend on threshold, the SROC curve is symmetric and can be described by a constant diagnostic odds ratio (DOR). The DOR is a single measure of test accuracy defined as the ratio of the odds of positivity in those who have the target condition relative to the odds of positivity in those without the condition. Therefore, a test with high TPR and low FPR will have a high DOR. This SROC approach is a fixed effect method in which variation is attributed solely to threshold effect and sampling error. The approach has methodological limitations which lead to inaccurate standard errors, thus rendering formal statistical inference invalid.^10,13^ Similar to the DerSimonian and Laird approach, zero cell corrections may be required.

### 2.3 Hierarchical models

Hierarchical models (also known as mixed or multilevel models) take into account correlation between sensitivity and specificity across studies while also allowing for variation in test performance between studies through the inclusion of random effects. The two main approaches – the bivariate model and the HSROC model – differ in parameterizations, but the models are mathematically equivalent when no covariates are included.^19^ The choice of approach is often determined by variation in the thresholds reported in the included studies and the focus of inference – a summary point or a SROC curve.

#### 2.3.1 Bivariate random effects model

van Houwelingen et al.^20^ proposed a bivariate approach to meta-analysis that was adapted by Reitsma et al.^1^ for test accuracy meta-analysis. This bivariate model is a linear mixed model that enables joint analysis of sensitivity and specificity and takes the form

(2) $$(\mu_{\mathrm{Ai}}\mu_{\mathrm{Bi}})∼N((\mu_{A}\mu_{B}),\sum_{\mathrm{AB}})\text{ with }\sum_{\mathrm{AB}}=(\sigma_{A}^{2}\sigma_{\mathrm{AB}}\sigma_{\mathrm{AB}}\sigma_{B}^{2})$$

The model assumes a bivariate normal distribution with mean $\mu_{A}$ and variance $\sigma_{A}^{2}$ for the logit sensitivities, mean $\mu_{B}$ and variance $\sigma_{B}^{2}$ for the logit specificities and $\sigma_{\mathrm{AB}}$the covariance between $\mu_{\mathrm{Ai}}$and $\mu_{\mathrm{Bi}}$across studies. Instead of the covariance, the model can be parameterized using the between study correlation, $\rho_{\mathrm{AB}}$. Therefore, the bivariate model without a covariate has the following five parameters: $\mu_{A}$*,* $\mu_{B}$_,_ $\sigma_{A}^{2}$, $\sigma_{B}^{2}$ and $\sigma_{\mathrm{AB}}$(or $\rho_{\mathrm{AB}}$). Chu and coworkers^7,12^ have shown that a binomial likelihood should be used for modelling within study variability (especially when data are sparse) as follows:

(3) $$y_{\mathrm{Ai}}∼\text{Binomial}(n_{\mathrm{Ai}},g^{-1}(\mu_{\mathrm{Ai}})),y_{\mathrm{Bi}}∼\text{Binomial}(n_{\mathrm{Bi}},g^{-1}(\mu_{\mathrm{Bi}}))$$

where $y_{\mathrm{Ai}}$ and $y_{\mathrm{Bi}}$ represent the number of true positives and true negatives, $n_{\mathrm{Ai}}$ and $n_{\mathrm{Bi}}$ the number of diseased and non-diseased subjects and $g^{-1}(\mu_{\mathrm{Ai}})$ and $g^{-1}(\mu_{\mathrm{Bi}})$ the sensitivity and specificity in the *i*th study, respectively. The logit link g(.) is commonly used but other link functions can be applied.^12,13^ The random effects also follow a bivariate normal distribution in this generalized linear mixed model. If this bivariate model is simplified by assuming the covariance or correlation is zero (i.e. an independent variance–covariance structure), the model reduces to two univariate random effects logistic regression models (UREMs) for sensitivity and specificity.

#### 2.3.2 HSROC model

The Rutter and Gatsonis HSROC model represents a general framework for meta-analysis of test accuracy studies and can be viewed as an extension of the Moses SROC approach in which the TPR and FPR for each study are modelled directly.^21^ The HSROC model is a nonlinear generalized mixed model and takes the form

(4) $$\text{logit}(\pi_{\mathrm{ij}})=(\theta_{i}+\alpha_{i}{\mathrm{dis}}_{\mathrm{ij}})\text{exp}(-\beta {\mathrm{dis}}_{\mathrm{ij}})$$

where $\pi_{\mathrm{ij}}$ is the proportion of test positives, true or false positives depending on disease status. Disease status is represented by ${\mathrm{dis}}_{\mathrm{ij}}$ which is coded −0.5 for the non-diseased (*j* = 0) and 0.5 for the diseased group (*j* = 1) in the *i*th study. The implicit threshold $\theta_{i}$ (threshold parameter or positivity criteria) and diagnostic accuracy $\alpha_{i}$ (accuracy parameter) for each study are modelled as random effects with independent normal distributions $\theta_{i}∼N(\Theta ,\sigma_{\theta }^{2})$ and $\alpha_{i}∼N(\Lambda ,\sigma_{\alpha }^{2})$, respectively. The model also includes a shape or scale parameter β which enables asymmetry in the SROC curve by allowing accuracy to vary with implicit threshold. Therefore, the SROC curve is symmetric if β=0 or asymmetric if β≠0. Each study contributes a single point in ROC space and so the estimation of β requires information from all studies included in the meta-analysis. Thus β is modelled as a fixed effect. The HSROC model has the following five parameters: Λ, Θ, β, $\sigma_{\alpha }^{2}$ and $\sigma_{\theta }^{2}$. The model reduces to a fixed effect model if $\sigma_{\alpha }^{2}=0$ and $\sigma_{\theta }^{2}=0$. Other specifications for SROC curves based on functions of the bivariate model have been proposed^10,22^ but in this paper we focus only on the more established and commonly used Rutter and Gatsonis model.

## 3 Motivating examples

### 3.1 Non-contrast computed tomography for diagnosing appendicitis

**Figure 1.**
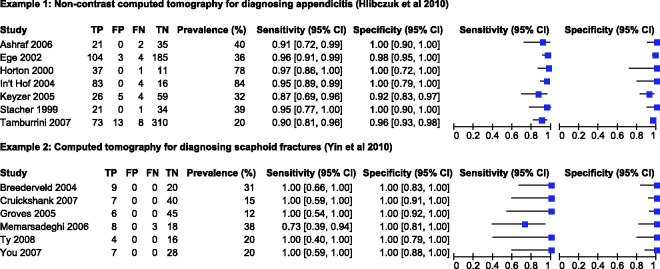
Forest plot of sensitivity and specificity estimates from studies included in the two motivating examples. FN: false negative; FP: false positive; TN: true negative; TP: true positive.

Hlibczuk et al.^23^ reviewed the diagnostic accuracy of non-contrast computed tomography (CT) for emergency department evaluation of adults with suspected appendicitis. Seven studies, evaluating 1060 patients of whom 389 had appendicitis, were included in the review. The prevalence of appendicitis in the studies ranged from 20% to 84%, with a median of 39%. The forest plot (Figure 1) shows between study variation in the sensitivities and specificities, though specificity was perfect (100%) in four studies. The authors attempted to fit the bivariate model in SAS but the model failed to converge.

### 3.2 CT for diagnosing scaphoid fractures

Yin et al.^24^ assessed the diagnostic accuracy of CT for diagnosing suspected scaphoid fractures. Six studies, evaluating 211 patients of whom 44 had a scaphoid fracture, were included in the review. The prevalence of scaphoid fractures in the studies ranged from 12% to 38%, with a median of 20%. Figure 1 shows the estimates of sensitivity and specificity with almost no between study variation; five of the six studies reported 100% sensitivity while all studies reported 100% specificities. The authors pooled sensitivity, specificity and the DOR using a random effects model (method not specified).

### 3.3 Results from reanalysis of the two example datasets

We reanalyzed the two datasets by fitting univariate, bivariate and HSROC models using the NLMIXED procedure in the SAS software package (version 9.2; SAS Institute, Cary, NC). UREMs for sensitivity and specificity were simultaneously obtained by setting the covariance parameter in a bivariate generalized linear mixed model equal to zero. This is equivalent to assuming an independent variance–covariance structure. Additional summary measures such as likelihood ratios and DORs were produced using the ESTIMATE statement within NLMIXED. The ESTIMATE statement computes additional estimates as a function of parameter values and produces standard errors and confidence intervals (CIs) using the delta method. Despite numerous attempts with different starting values and optimization algorithms, the bivariate model failed to converge for both datasets. In addition, the HSROC model containing all five parameters failed to converge for the scaphoid fractures dataset. The models fitted and results obtained for both datasets are summarised in Table 1. For the appendicitis dataset, the complete HSROC model successfully converged and produced reliable estimates only when boundary constraints (*σ*^2 ^≥ 0) were specified for $\sigma_{\alpha }^{2}$ and $\sigma_{\theta }^{2}$; the boundary constraint for $\sigma_{\theta }^{2}$ was activated (estimation truncated at zero) and the between study correlation was estimated as +1. This is due to the maximum likelihood estimator truncating the between-study covariance matrix on the boundary of its parameter space.^15^ A bivariate model with a correlation of +1 corresponds to an HSROC model with $\sigma_{\theta }^{2}$ truncated at zero, and a correlation of −1 corresponds to an HSROC model with $\sigma_{\alpha }^{2}$ truncated at zero.

**Table 1.** Summary accuracy measures obtained from different meta-analytic models applied to the two motivating examples.

Meta-analytic model	Sensitivity (95% CI)	Specificity (95% CI)	LR + (95% CI)	LR − (95% CI)	DOR (95% CI)
Non-contrast CT for appendicitis (*n* = 7)					
Univariate fixed effect logistic regression	93.8 (90.6, 96.0)	96.9 (95.1, 98.0)	30 (19, 48)	0.06 (0.04, 0.10)	471 (244, 907)
Univariate random effects logistic regression	93.8 (90.8, 95.9)	97.4 (93.7, 99.0)	37 (14, 93)	0.06 (0.04, 0.10)	579 (205, 1635)
Bivariate random effects model	NE	NE	NE	NE	NE
Complete HSROC	94.1 (88.5, 97.0)	97.8 (92.6, 99.4)	42 (12, 148)	0.06 (0.03, 0.12)	700 (130, 3771)
Symmetric HSROC	94.1 (88.2, 97.2)	97.5 (94.1, 99.0)	38 (15, 93)	0.06 (0.03, 0.13)	628 (149, 2657)
Fixed accuracy	93.8 (90.1, 96.2)	96.9 (94.7, 98.2)	30 (18, 51)	0.06 (0.04, 0.10)	471 (223, 995)
Fixed threshold	94.1 (88.9, 96.9)	97.8 (93.0, 99.3)	42 (13, 140)	0.06 (0.03, 0.12)	701 (141, 3485)
Fixed accuracy and threshold	93.8 (90.6, 96.0)	96.9 (95.1, 98.0)	30 (19, 48)	0.06 (0.04, 0.10)	471 (244, 907)
Symmetric fixed accuracy and threshold	93.8 (90.6, 96.0)	96.9 (95.1, 98.0)	30 (19, 48)	0.06 (0.04, 0.10)	471 (244, 907)
CT for scaphoid fractures (*n* = 6)					
Univariate fixed effect logistic regression	93.2 (78.8, 98.1)	100	2.26E + 07 (NE)	0.07 (0.02, 0.23)	3.31E + 08 (NE)
Univariate random effects logistic regression	99.0 (3.7, 100)	100	3.25E + 07 (NE)	0.01 (3.94E − 06, 24)	3.34E + 09 (NE)
Bivariate random effects	NE	NE	NE	NE	NE
Complete HSROC	99.1 (2.2, 100)	100	2.07E + 09 (NE)	0.01 (2.14E − 06, 41)	2.21E + 11 (NE)
Symmetric HSROC	98.6 (12.9, 100)	100 (0, 100)	54,762 (1.29E − 05, 2.33E + 14)	0.01 (3.36E − 05, 6.11)	3,818,334 (0.0001, 1.33E + 17)
Fixed accuracy	99.0 (6.4, 100)	100	1.59E + 11 (NE)	0.01 (7.03E − 06, 13)	1.64E + 13 (NE)
Fixed threshold	99.0 (6.4, 100)	100	2.37E + 09 (NE)	0.01 (7.03E − 06, 13)	2.43E + 11 (NE)
Fixed accuracy and threshold	93.2 (78.8, 98.1)	100	7.80E + 09 (NE)	0.07 (0.02, 0.23)	1.14E + 11 (NE)
Symmetric fixed accuracy and threshold	93.2 (78.8, 98.1)	100	2.27E + 07 (NE)	0.07 (0.02, 0.23)	3.34E + 08 (NE)

*n*: number of studies in the meta-analysis, NE: not estimable.

Since the maximum likelihood estimation problems encountered with the bivariate model are most likely due to boundary estimation of the variance and/or covariance parameters, we attempted plotting the profile log likelihood for the covariance parameter (maximized with respect to the other 4 parameters). We were unable to produce a plot for the scaphoid fracture example because the bivariate model failed even with fixed values for the covariance. This is unsurprising since there was almost no between study variation in sensitivity and specificity.

**Figure 2.**
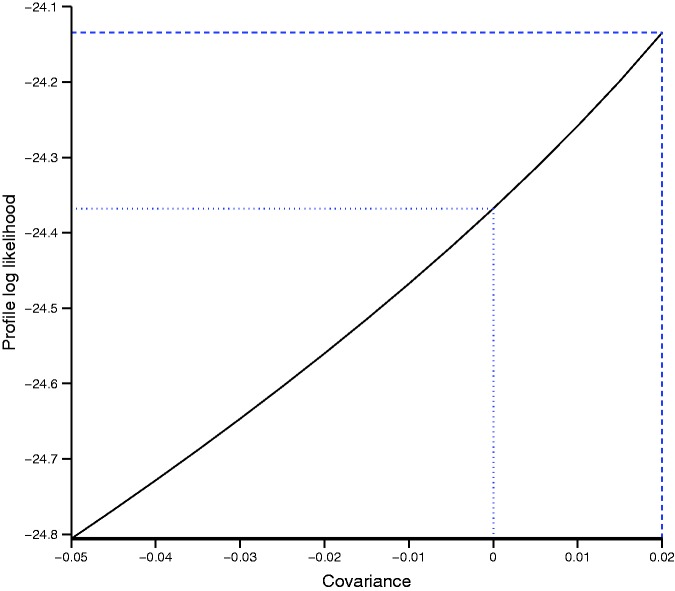
Profile log-likelihood function of the covariance parameter in the bivariate model applied to the appendicitis example.

Figure 2 shows the profile log likelihood for the covariance parameter for the appendicitis example. The likelihood is flat with very little change in the profile log likelihood. The maximum of the profile log likelihood was achieved at a covariance of 0.02 (dashed line). For covariances above 0.02, the bivariate model failed to converge or was unstable, but values between −0.05 and 0.02 appear to be supported by the data. The dotted line shows the value of the log likelihood for a covariance of zero, i.e. independence between sensitivity and specificity. This suggests that UREMs would be appropriate for pooling sensitivity and specificity in this example.

The two examples illustrate the problem of model convergence, poor parameter estimation and the need for simpler models. There were only subtle differences in summary estimates and 95% CIs for sensitivity, specificity and the negative likelihood ratio between models fitted to the appendicitis dataset. In contrast, clear differences were observed for the positive likelihood ratio and the DOR. For the scaphoid fractures dataset, there were differences in summary estimates and 95% CI for sensitivity and specificity from the univariate fixed effect model and the HSROC models with both fixed accuracy and threshold parameters compared to the other models. These examples show that results can differ between models, and the differences may not be negligible. Therefore, the identification of simpler meta-analytic methods that give valid answers in situations where complex models fail is of practical importance.

## 4 Simulation study

### 4.1 Simulation methods

We conducted a simulation study to compare the performance of a UREM and the HSROC model with various simplifications (by removing model parameters). Given the mathematical equivalence of the HSROC and bivariate models when no covariate is included, there was no need to examine the performance of both models. We chose the HSROC model because it has greater flexibility for introducing model parsimony by dropping parameters than the bivariate model.^19^ Since several authors^7–9,15^ have shown that approximate methods for modelling within study variability are biased, we only investigated methods that use a binomial likelihood. The specifications for the scenarios were devised to replicate realistic situations encountered in meta-analysis of diagnostic accuracy studies. We investigated the effect of these factors: 1) number of studies; 2) magnitude of diagnostic accuracy (DOR); 3) prevalence of disease; 4) between study variation in accuracy and threshold; and 5) asymmetry in the SROC curve. We modified the simulation approach used in a previous study^25^ to define the simulation scenarios and generate the simulated datasets as described below.

#### 4.1.1 Generation of simulated data

To determine diagnostic accuracy, we used the standardised distance between the means $\mu_{1}$ and $\mu_{2}$ (where $\mu_{2}>\mu_{1}$) of the logistic distributions for non-diseased and diseased, respectively. We selected the diagnostic threshold, *t*, as the average of the means of the two distributions, i.e. $t=(\mu_{1}+\mu_{2})/2$. If the two distributions have different standard deviations ($\sigma_{1}\neq \sigma_{2}$), sensitivity ≠ specificity at *t* and the SROC curve has an asymmetric shape. The DOR at *t* can be calculated as follows:

(5) $$\text{DOR}=\text{exp}[\sqrt{\frac{\pi^{2}}{3}}(\frac{\mu_{2}-t}{\sigma_{2}}-\frac{\mu_{1}-t}{\sigma_{1}})]$$

The sensitivity and specificity at *t* can be obtained using the following:

(6) $$\text{Sensitivity}=\frac{\text{exp}[\sqrt{\frac{\pi^{2}}{3}}(\frac{\mu_{2}-t}{\sigma_{2}})]}{1-\text{exp}[\sqrt{\frac{\pi^{2}}{3}}(\frac{\mu_{2}-t}{\sigma_{2}})]},\text{Specificity}=1-\frac{\text{exp}[\sqrt{\frac{\pi^{2}}{3}}(\frac{\mu_{1}-t}{\sigma_{1}})]}{1+\text{exp}[\sqrt{\frac{\pi^{2}}{3}}(\frac{\mu_{1}-t}{\sigma_{1}})]}$$

When the distributions of test results for the diseased and non-diseased have the same standard deviation ($\sigma_{1}=\sigma_{2}=\sigma$), sensitivity = specificity at *t* and the SROC curve has a symmetric shape. For scenarios where $\sigma_{1}=\sigma_{2}=\sigma$, we investigated values of diagnostic accuracy that correspond to the following:

1. $(\mu_{2}-\mu_{1})/\sigma =2$ (log DOR = 3.63, DOR = 38; sensitivity = specificity = 0.86);
2. $(\mu_{2}-\mu_{1})/\sigma =3$ (log DOR = 5.44, DOR = 231; sensitivity = specificity = 0.94)

For scenarios where $\sigma_{2}=2\sigma_{1}$, using the same $\mu_{2}$ and $\mu_{1}$ as in (1) and (2) above, the DOR of 38 reduces to 15 (sensitivity = 0.71 and specificity = 0.86) and the DOR of 231 reduces to 59 (sensitivity = 0.80 and specificity = 0.94).

We investigated meta-analyses with different number of studies (*k* = 5, 10, 20). The size of a study in each meta-analysis, *n_j_*, was randomly sampled from a uniform distribution, *U*(20,200). We varied *n_j_* between 20 and 200 because diagnostic accuracy studies are often small in size.^17,26^ Given an underlying prevalence *p*, individuals within each study were randomly classified as diseased or non-diseased, and assigned a continuous test result value, *x*, which was randomly sampled from the logistic distributions. For each study, we used *t* to determine the outcome of an individual's test result; positive if $x_{\mathrm{ij}}>t$, or negative if $x_{\mathrm{ij}}\leq t$. To create the 2 x 2 table for each study, individuals were then classified as true positives, false negatives, false positives or true negatives based on test result and disease status.

To begin we assumed zero between study variation in both accuracy and threshold. We then introduced between study variation in diagnostic accuracy by adding a value τ sampled from a normal distribution with zero mean and standard deviation 0.3$\sigma_{1}$. This value was added to the difference in means ($\mu_{2}-\mu_{1}$) for each study. We introduced between study variation in diagnostic threshold by also sampling from a normal distribution with the average threshold *t* as the mean and standard deviation 0.3$\sigma_{1}$. We generated 10,000 independent meta-analysis datasets for each scenario to enable precise estimation of model performance even if a large proportion of models fail to converge. If all 10,000 datasets for each scenario successfully converged, they will give a standard error of 0.0022 for the estimation of 95% CI coverage probability.^27^ However if only 1000 datasets converged, the standard error will be 0.0069. The datasets were created using Stata version 10.1 (Stata-Corp, College Station, TX). Table 2 summarises the different scenarios investigated. The meta-analysis dataset for the base scenario for each DOR contained five studies with an underlying prevalence of 5% and no heterogeneity in accuracy or threshold.

**Table 2.** Scenarios evaluated in the simulation.^a^

Scenario	Prevalence (%)	DOR	Heterogeneity in accuracy and threshold	Asymmetry in SROC curve
1–3	5	38	No	No
4–6	25	38	No	No
7–9	50	38	No	No
10–12	5	38	Yes	No
13–15	25	38	Yes	No
16–18	50	38	Yes	No
19–21	5	231	No	No
22–24	25	231	No	No
25–27	50	231	No	No
28–30	5	231	Yes	No
31–33	25	231	Yes	No
34–36	50	231	Yes	No
37–39	5	15	Yes	Yes
40–42	25	15	Yes	Yes
43–45	50	15	Yes	Yes
46–48	5	59	Yes	Yes
49–51	25	59	Yes	Yes
52–54	50	59	Yes	Yes

a Each subset of 3 scenarios corresponds to 5, 10 and 20 studies.

#### 4.1.2 Meta-analytic models fitted to each dataset

Throughout the rest of this paper, we refer to an HSROC model that contained all five parameters as a complete HSROC model. We fitted the following seven models to each meta-analysis dataset.

1. UREM – includes $\mu_{A}$ and $\sigma_{A}^{2}$ for the logit sensitivities, and $\mu_{B}$ and $\sigma_{B}^{2}$ for the logit specificities. Note this is a simplification of the bivariate generalized mixed model achieved by setting the covariance or correlation parameter to zero (see section 2.3.1). For brevity, from here on we will refer to this model simply as the univariate random effects model.
2. Complete HSROC model – includes all five parameters Λ, Θ, β, $\sigma_{\alpha }^{2}$ and $\sigma_{\theta }^{2}$
3. Symmetric HSROC model – includes Λ, Θ, $\sigma_{\alpha }^{2}$ and $\sigma_{\theta }^{2}$
4. HSROC model with fixed threshold – includes Λ, Θ, β and $\sigma_{\alpha }^{2}$
5. HSROC model with fixed accuracy – includes Λ, Θ, β and $\sigma_{\theta }^{2}$
6. HSROC model with fixed accuracy and threshold – includes Λ, Θ and β (allows for asymmetry in the SROC curve)
7. Symmetric HSROC model with fixed accuracy and threshold parameters – includes only two parameters Λ and Θ

As shown by Harbord et al.,^19^ the five parameters of the bivariate model can be expressed in terms of those of the HSROC model as follows:

(7) $$\mu_{A}=\text{exp}(-\frac{\beta }{2})(\Theta +\frac{\Lambda }{2}),\mu_{B}=-\text{exp}(\frac{\beta }{2})(\Theta -\frac{\Lambda }{2})$$

(8) $$\sigma_{A}^{2}=\text{exp}(-\beta )(\sigma_{\theta }^{2}+\frac{1}{4}\sigma_{\alpha }^{2}),\sigma_{B}^{2}=\text{exp}(\beta )(\sigma_{\theta }^{2}+\frac{1}{4}\sigma_{\alpha }^{2}),\sigma_{\mathrm{AB}}=-(\sigma_{\theta }^{2}-\frac{1}{4}\sigma_{\alpha }^{2})$$

For the fixed accuracy threshold and symmetric fixed accuracy threshold models, $\sigma_{\alpha }^{2}=0$ and $\sigma_{\theta }^{2}=0$. Thus $\sigma_{A}^{2}=0$, $\sigma_{B}^{2}=0$ and$\sigma_{\mathrm{AB}}=0$, and both models are equivalent to simultaneously fitting two univariate fixed effect logistic regression models for sensitivity and specificity (see results for these models in Table 1). Henceforth, we refer to them as fixed effect models; the models can be considered a special case of the random effects models where the variances of the random effects are zero. We used the SAS NLMIXED procedure to fit each of the seven meta-analytic models because Stata does not have an inbuilt or user defined command for fitting non-linear generalized mixed models. Note that because of the mathematical relationship between the bivariate and HSROC model, it is possible in Stata to obtain estimates for the five parameters of the HSROC model using functions of parameters from the bivariate model fitted.^19^ We computed additional estimates by using the ESTIMATE statement. We computed the log DOR at the average operating point (summary sensitivity and specificity). This log DOR is exactly the same value as Λ if the SROC curve is symmetric.

#### 4.1.3 Facilitating convergence of hierarchical models

To aid convergence, we provided a wide range of starting values for model parameters by specifying a grid of points for a grid search of starting values. We used a quasi-Newton optimization technique (the NLMIXED default) because it provides an appropriate balance between computation speed and stability (SAS Institute Inc. SAS OnlineDoc® 9.1.3. Cary, NC, 2004). To prevent estimation of negative variances and to reduce computational problems, we specified boundary constraints (*σ*^2 ^≥ 0) for the variance parameters in the models. To reduce the number of models that failed to converge, we refitted models by trying a new set of starting values and/or changing the optimization technique to a Newton-Raphson technique. To obtain a new set of starting values, we fitted a model with no random effects and used the new parameter estimates together with the original grid of points for the variance parameters. Thus for some datasets, we made up to four attempts to fit a hierarchical model.

#### 4.1.4 Assessment of model convergence and stability

Because a model that meets a convergence criterion may be unstable or have missing standard errors due to issues with model identifiability, we assessed convergence in two stages. First, we checked whether the convergence criterion was met and also whether the additional estimates defined in the ESTIMATE statements were produced. Second, because standard errors are computed from the final Hessian matrix, we calculated eigenvalues of the Hessian to detect if there were problems. At a true minimum, eigenvalues will all be positive, i.e. positive definite. Therefore, for convergence to be deemed successful, the model had to meet the convergence criterion, produce additional estimates, and the Hessian had to be positive definite.

#### 4.1.5 Assessment of performance of meta-analytic models

We assessed performance of the methods by examining estimates of the following measures of diagnostic accuracy: log DOR, logit sensitivity and logit specificity. We assessed estimability as the percentage of meta-analyses that successfully converged and the percentage where the between study correlation was not estimated as −1 or +1. We computed the latter for only the complete HSROC model. For each scenario, we used only the results from meta-analyses that successfully converged as defined above to calculate (a) the difference between the average parameter estimate and the true parameter value to determine bias; (b) the average standard error and mean square error (MSE incorporates both bias and variability) to assess model accuracy; and (c) the coverage of the 95% CIs by computing the percentage of meta-analyses for which the true parameter value was within the 95% CI.

### 4.2 Simulation results

Altogether we explored 54 scenarios. We can only show results for the log DOR in this article but results for logit sensitivity and logit specificity are briefly mentioned. Because homogeneous accuracy and threshold are the exception rather than the norm for meta-analysis of test accuracy studies, to illustrate key findings, we present results mainly for scenarios with heterogeneity at a DOR of 231 (sparse data are of interest and zero false positives and/or false negatives are more likely to occur when diagnostic accuracy is high).

#### 4.2.1 Estimability

Zero cells occurred frequently especially when diagnostic accuracy was high (Table 3). Convergence rates were higher for the complete HSROC model in scenarios with heterogeneity compared to scenarios without heterogeneity. This is likely due to the inclusion of heterogeneity parameters in the HSROC model that become problematic to estimate when the true heterogeneity is zero. Convergence increased with increasing number of studies and prevalence, and with decreasing diagnostic accuracy. Convergence decreased in scenarios with asymmetry in the SROC curve (data not shown). Across scenarios, non-convergence and problems with model identifiability were more common with the complete HSROC and fixed threshold models compared to the other hierarchical models (Table 4); the symmetric fixed accuracy threshold model always converged. The complete HSROC model often poorly estimated the correlation between the logit transformed sensitivities and specificities as +1 or −1 (Table 3); estimation as −1 occurred much more frequently than +1. The correlation was more likely to be estimated between −1 and +1 when there was heterogeneity in accuracy and threshold, greater prevalence of disease and more studies in a meta-analysis.

**Table 3.** Convergence and estimability of the complete HSROC model applied to 10,000 datasets in 36 different scenarios.^a^

DOR	*N*	Prevalence (%)	No heterogeneity in accuracy and threshold					Heterogeneity in accuracy and threshold				
			Meta- analyses with a zero cell^b^ (%)	Successful model fit (positive definite) (%)	% ρ^AB =−1	% ρ^AB = +1	% ρ^AB ≠ −1 or +1	Meta- analyses with a zero cell^b^ (%)	Successful model fit (positive definite) (%)	% ρ^AB = −1	% ρ^AB = +1	% ρ^AB ≠ −1 or +1
38	5	5	48	18	14	2.6	1.8	50	36	21	0.6	14
38	5	25	50	18	15	1.7	1.6	51	54	31	0.2	22
38	5	50	52	18	15	1.4	2.0	53	60	34	0.2	26
38	10	5	60	25	17	3.7	4.4	60	52	21	0.2	31
38	10	25	65	24	18	2.4	3.8	67	77	24	0.0	54
38	10	50	72	25	18	2.6	4.1	73	85	24	0.0	61
38	20	5	75	32	20	5.8	6.4	77	70	20	0.0	50
38	20	25	77	30	20	3.7	6.3	78	93	12	0.0	82
38	20	50	82	28	20	3.1	5.8	84	97	10	0.0	88
231	5	5	96	18	11	5.7	1.4	97	30	18	2.4	10
231	5	25	97	21	14	5.0	1.6	98	43	29	2.1	13
231	5	50	99	21	15	4.7	1.9	99	48	31	2.3	14
231	10	5	99	23	13	7.7	3.0	99	41	21	1.3	19
231	10	25	99	29	17	7.6	4.1	100	62	29	0.9	33
231	10	50	100	29	18	6.7	4.1	100	71	32	0.9	39
231	20	5	100	29	15	9.7	4.8	100	54	20	0.3	34
231	20	25	100	35	20	9.2	6.2	100	80	23	0.2	57
231	20	50	100	35	20	9.2	6.5	100	88	22	0.1	66

ρ^AB: estimated between study correlation; DOR: diagnostic odds ratio; *N*: number of studies.

a All results are presented as percentages and are based on 10,000 meta-analysis datasets.

b The percentage of meta-analyses out of 10,000 where at least one study included a zero cell.

**Table 4.** Performance of all meta-analytic models in estimating the log DOR for scenarios with a DOR of 231.

Studies	Heterogeneity^a^	Meta-analytic model	5% prevalence				25% prevalence				50% prevalence			
			*N*	Bias (%)^b^	MSE	Coverage (%)	*N*	Bias (%)	MSE	Coverage (%)	*N*	Bias (%)	MSE	Coverage (%)
5	No	Complete HSROC	1767	13.5	6.24	98.9	2086	4.20	0.41	98.8	2114	3.27	0.23	97.7
		Symmetric HSROC	1276	4.32	4.26	95.5	2753	2.28	0.26	97.1	4179	2.58	0.20	97.4
		FA	1932	36.2	31.9	98.7	2228	4.86	0.59	98.5	2232	3.64	0.26	97.4
		FT	1792	37.8	32.6	98.9	2174	4.77	0.63	99.3	2288	3.26	0.27	98.9
		FAT	9798	30.1	28.0	97.0	9556	1.82	0.37	95.9	4722	1.24	0.17	94.5
		SFAT	10,000	37.0	40.7	88.5	10,000	1.69	0.42	95.8	10,000	1.19	0.15	95.5
		UREM	3173	11.2	5.67	98.5	2869	3.69	0.40	97.5	2883	2.71	0.22	97.4
5	Yes	Complete HSROC	3020	40.2	41.6	97.6	4339	2.43	0.57	95.8	4772	1.26	0.30	94.4
		Symmetric HSROC	3442	20.6	20.6	94.2	6331	0.76	0.33	93.6	7594	0.58	0.25	93.3
		FA	5490	40.6	42.6	97.1	6222	2.97	0.82	95.4	6331	1.57	0.31	93.9
		FT	2976	51.9	51.4	98.2	2266	3.66	1.12	97.7	2171	2.26	0.36	97.9
		FAT	9691	22.3	22.9	91.8	9288	−2.16	0.66	85.1	4833	−3.18	0.30	79.4
		SFAT	10,000	28.3	33.0	84.2	10,000	−2.38	0.77	84.6	10,000	−3.23	0.30	80.7
		UREM	5833	11.8	6.53	97.8	6311	2.01	0.43	96.8	6573	1.10	0.30	96.4
10	No	Complete HSROC	2325	7.70	0.94	99.0	2903	2.42	0.15	97.2	2862	1.90	0.10	97.0
		Symmetric HSROC	1924	1.70	0.37	97.3	3581	1.60	0.11	96.4	5383	1.50	0.09	96.9
		FA	2175	11.0	4.52	98.7	2569	2.58	0.15	97.3	2719	1.96	0.10	96.4
		FT	2177	10.6	4.55	98.9	2670	2.44	0.15	98.3	2666	1.72	0.10	98.2
		FAT	9881	5.55	3.45	96.9	9594	0.71	0.09	95.5	4596	0.55	0.07	95.5
		SFAT	10,000	6.40	4.96	95.9	10,000	0.62	0.09	95.4	10,000	0.51	0.07	95.4
		UREM	5612	6.59	1.02	98.4	5417	1.71	0.12	97.0	5311	1.30	0.08	96.7
10	Yes	Complete HSROC	4129	9.55	4.58	98.1	6248	0.93	0.16	95.2	7136	0.61	0.13	94.5
		Symmetric HSROC	5895	2.72	1.89	95.8	8488	0.20	0.14	93.7	9387	0.35	0.12	93.4
		FA	6772	9.14	5.04	96.9	7776	0.45	0.15	93.6	8088	0.30	0.12	92.1
		FT	2759	10.8	6.67	98.0	1840	0.60	0.17	96.9	1579	0.27	0.13	97.5
		FAT	9775	−0.31	2.42	88.0	9301	−4.19	0.23	77.3	4765	−4.62	0.24	71.4
		SFAT	10,000	0.15	3.34	87.0	10,000	−4.38	0.24	76.7	10,000	−4.45	0.23	72.2
		UREM	8302	5.30	1.42	97.4	8942	0.46	0.16	96.6	9237	0.36	0.12	97.1
20	No	Complete HSROC	2915	4.87	0.40	99.4	3513	1.62	0.07	97.0	3513	1.28	0.05	95.9
		Symmetric HSROC	2654	1.16	0.16	96.1	4359	1.01	0.05	96.1	6149	1.04	0.04	96.3
		FA	2425	4.77	0.44	98.5	2969	1.60	0.06	96.5	3076	1.19	0.04	95.7
		FT	2439	4.47	0.39	99.1	2888	1.53	0.07	97.6	2996	1.23	0.05	97.1
		FAT	9917	1.25	0.17	95.7	9615	0.37	0.04	95.2	4528	0.34	0.03	94.7
		SFAT	10,000	1.21	0.17	95.7	10,000	0.34	0.04	95.1	10,000	0.29	0.03	94.9
		UREM	8094	3.64	0.33	97.1	7930	1.03	0.05	96.5	7963	0.83	0.04	96.1
20	Yes	Complete HSROC	5406	3.36	0.39	97.2	8011	0.43	0.07	95.5	8843	0.14	0.06	94.3
		Symmetric HSROC	8040	0.51	0.17	95.0	9679	0.14	0.07	94.1	9905	0.06	0.06	93.7
		FA	7767	1.38	0.33	95.3	8930	−0.34	0.07	92.4	9179	−0.39	0.06	90.7
		FT	2054	0.95	0.24	95.9	992	−0.68	0.07	95.8	781	−0.95	0.06	95.6
		FAT	9758	−4.37	0.30	81.7	9293	−4.88	0.20	66.0	4559	−5.19	0.21	58.3
		SFAT	10,000	−4.46	0.30	81.4	10,000	−5.01	0.21	64.4	10,000	−5.12	0.21	57.6
		UREM	9455	1.64	0.29	96.4	9869	0.11	0.07	97.4	9951	0.04	0.06	97.2

DOR: diagnostic odds ratio; FA: fixed accuracy HSROC model; FAT: fixed accuracy and threshold HSROC model; FT: fixed threshold HSROC model; MSE: mean square error; *N*: number of meta-analyses out of 10,000 where hierarchical models successfully converged; SFAT: symmetric fixed accuracy and threshold HSROC model; UREM: univariate random effects logistic regression model.

a Heterogeneity in accuracy and threshold.

b Bias is presented as a percentage of the true value of the log diagnostic odds ratio.

#### 4.2.2 Bias

In the base scenario for a DOR of 231, the symmetric HSROC model gave the least percentage bias for the DOR (4.32%); bias was highest for the fixed threshold (37.8%) and fixed accuracy (36.2%) models (Table 4). These rankings were consistent as the number of studies increased. As prevalence increased, the two fixed effect models became the least biased while the fixed accuracy model remained the most biased.

When heterogeneity was introduced, each of the seven models produced the largest bias for the DOR at the lowest prevalence, though the univariate random effects model gave the least biased DOR. For all models, bias decreased as prevalence and the number of studies increased. However, the decrease in bias resulted in a change from overestimation to underestimation for the two fixed effect models. For bias in the estimates of sensitivity, we observed results similar to those of the DOR, but the relationship with prevalence was reversed for bias in the estimates of specificity (data not shown). Bias in specificity was very small compared to that of the DOR or sensitivity. For the three measures, in scenarios with heterogeneity and asymmetry in the SROC curve, bias was lower than in the corresponding symmetric model.

#### 4.2.3 Model accuracy

A MSE of zero indicates that the model estimated the parameter of interest with perfect accuracy, i.e. no bias and no variability in the estimation. The MSE of the DOR was highest for the symmetric fixed accuracy threshold model (40.7) but lowest for the symmetric HSROC model (4.26) in the base scenario (Table 4). At higher prevalence, the two fixed effect models had the lowest MSE. For all models, the MSE of the DOR decreased as the number of studies and prevalence increased. When heterogeneity was introduced, the univariate random effects model had the lowest MSE at 5% prevalence but the symmetric HSROC model had slightly lower MSE than the univariate random effects model at higher values of prevalence. As the number of studies and prevalence increased, the MSE for all models decreased and became almost identical except for those of the two fixed effect models. Results for sensitivity were similar to those for the DOR. The MSE for specificity was generally very low and increased slightly with increasing prevalence. For the asymmetric SROC curve scenarios, the findings for the three measures were similar to those of the corresponding symmetric scenarios.

#### 4.2.4 Coverage

For a DOR of 231, the symmetric HSROC models gave the best coverage of the 95% CIs for estimation of the DOR (95.5%) in the base scenario. With the exception of the symmetric fixed accuracy threshold model, all models were conservative as shown by coverage greater than 95%. The coverage of 88% for the symmetric fixed accuracy threshold model implied over-confidence in the estimates but coverage increased as prevalence or the number of studies increased. In contrast, introduction of heterogeneity led to very poor coverage for the two fixed effect models with coverage becoming lower as prevalence increased. The univariate random effects model and symmetric HSROC model often showed good coverage, although the latter tended to show under-coverage as prevalence increased. For sensitivity, the results were comparable to those of the DOR. Across all models, coverage was low for specificity when there was heterogeneity unlike scenarios without heterogeneity. The asymmetric SROC curve scenarios produced similar results to the symmetric SROC curve scenarios.

#### 4.2.5 Summary of simulation results and application to motivating examples

The following key points were observed:

- Hierarchical models are more likely to converge if there is heterogeneity in accuracy and threshold.
- Convergence is also affected by number of studies, prevalence and magnitude of diagnostic accuracy.
- Correlation between sensitivity and specificity across studies is often poorly estimated as +1 or −1.
- In the absence of heterogeneity, the two fixed effect models were the least biased with low MSE and good coverage properties for studies with moderate to high prevalence. The symmetric fixed accuracy threshold model may be of greater utility because it always converged. The symmetric HSROC model performed better than both fixed effect models when prevalence was low and there were few studies, but this finding was based on a convergence rate as low as 13%.
- When heterogeneity was present, the univariate random effects model and the symmetric HSROC model were often the least biased with low MSE and good coverage (however, there is a risk of selection bias in these results for scenarios with lower prevalence with smaller numbers of studies where as few as 34% of simulations converged).

In the simulation, the fixed threshold model often gave biased and imprecise results. However, for the appendicitis example, the fixed threshold model gave results similar to the complete HSROC model. The results can be explained by the fact that the estimation of $\sigma_{\theta }^{2}$ was truncated at zero in the complete model and so removing $\sigma_{\theta }^{2}$ from the HSROC model was appropriate in this example unlike in the simulation scenarios. The results in Table 1 indicate that while the univariate random effects model and symmetric HSROC model appear to be generally applicable when there is heterogeneity, other models like the fixed threshold or fixed accuracy can be considered if it is apparent the variance parameter for threshold or accuracy cannot be estimated.

For the scaphoid fractures example, the results of the simulation indicate that using a univariate fixed effect model (including the equivalent fixed accuracy threshold and symmetric fixed accuracy threshold models) was valid because there was no heterogeneity in the specificities (all six studies reported 100% specificity) and very limited heterogeneity in the sensitivities (five of the studies reported 100% sensitivity). Even for the fixed effect models, computation of the positive likelihood ratio and DOR were problematic because of the perfect specificity.

## 5 Discussion

In this study we simulated meta-analyses under a number of scenarios and evaluated hierarchical models for meta-analysis of diagnostic accuracy studies. Our findings indicate that simplifying hierarchical models is valid when there are few studies or sparse data. Our recommendations for selecting alternative models when bivariate or HSROC models fail to converge or converge but give unreliable estimates, are outlined in Box 1. If estimation of an average operating point (summary sensitivity and specificity) is of interest instead of a SROC curve, we recommend a univariate logistic regression approach with or without random effects depending on the extent to which sensitivity and/or specificity vary between studies. These methods are an appropriate alternative for obtaining independent summaries of sensitivity and specificity with CIs. However, joint inferences cannot be made about sensitivity and specificity through confidence and prediction regions around the average operating point. These regions account for correlation between sensitivity and specificity, and are useful for illustrating uncertainty around the average operating point and the extent of heterogeneity. If interest lies in the estimation of a SROC curve, the symmetric HSROC model or its fixed effect equivalent should be considered instead. The symmetric HSROC model is equivalent to fitting a bivariate model with an exchangeable covariance structure, where the variance of the random effects for the logit sensitivities is assumed to be the same as that of the logit specificities. In extreme situations with no heterogeneity and sparse data, such as the scaphoid fractures example, even the simplest models may fail to produce usable summary estimates.

**Box 1.** Recommendations for selecting alternative models when bivariate or HSROC models fail.^a^

**Plot the data**		
Visual inspection of forest plots and SROC plots may help to identify whether heterogeneity exists. For example, one may observe complete or near complete lack of variability between estimates of sensitivity and/or specificity, indicating no heterogeneity in one or both parameters (sensitivity and/or specificity equal to 100%), or conversely wide variability in observed estimates (e.g. non-overlapping confidence intervals) indicating large heterogeneity.		
**Analyses**		
Select a simpler hierarchical fixed effect or random effects model based on inference of interest (summary points or SROC curve), observation from the data plot, and previous output from the failed bivariate or HSROC model		
Note: when prevalence is very low and the number of studies is very small, there is potential for bias and the results of the meta-analysis should be interpreted with caution.		
	Focus of inference	
Heterogeneity	Summary point (summary sensitivity and specificity)	SROC curve
Variability in sensitivity and/or specificity between studies observed on the plot	Univariate random effects logistic regression models	Symmetric HSROC model
Minimal or no variability in sensitivity and/or specificity between studies observed on the plot	Univariate fixed effect logistic regression models^b^	Symmetric fixed accuracy and threshold model
A symmetric SROC curve can be described using the diagnostic odds ratio (exponent of the value of the accuracy parameter).		
Section 4.1.3 contains suggestions for facilitating convergence of hierarchical models.		

a Bivariate or HSROC models either failed to converge or converged (i.e. met the convergence criterion) but gave unreliable estimates (e.g. with no standard errors, or dependent on starting values).

b The symmetric fixed accuracy threshold model is equivalent to simultaneously fitting two univariate fixed effect logistic regression models for sensitivity and specificity.

Given the poor performance of simpler models like the fixed accuracy and fixed threshold models in the simulation, we urge meta-analysts to carefully explore their data and visually inspect forest plots and SROC plots before undertaking meta-analyses. Such preliminary analyses will provide an indication of the degree of heterogeneity and the pattern of scatter of the study points in ROC space. These analyses and the output from unstable or failed models should inform the approach for simplifying hierarchical models as shown by the appendicitis example. Although more complex and seldom used in practice, a Bayesian approach is an alternative to the maximum likelihood approach. In an empirical evaluation, both approaches were found to be similar although Bayesian methods suggested greater uncertainty (wide credible intervals) around the point estimates.^6^

A normal distribution is typically assumed for the random effects in hierarchical meta-analytic models; violation of this assumption may contribute to non-convergence. Heavy tailed distributions such as *t* or Cauchy distributions may be used instead of a normal distribution,^2,11^ but random effects are restricted to be normally distributed in SAS NLMIXED and Stata. A Bayesian approach allows alternative distributions though a normal distribution is often assumed in practice.^21^ As the models are often fitted using a maximum likelihood approach, our intention was to offer solutions within the hierarchical framework recommended for meta-analysis, using one of the software packages that have made meta-analysis of test accuracy studies more accessible to meta-analysts. A composite likelihood approach (implemented in R using the glmmML package) that offers some robustness to model misspecifications was recently proposed.^28^ Results from the simulation study where the composite likelihood method and the bivariate generalized mixed model were applied to data generated from a bivariate *t* distribution suggested the methods were insensitive to the heavy tailed distribution under the logit link function. We used only the logit link in our models.

Our simulations and application to motivating examples support and extend empirical evidence suggesting that univariate methods generate summary results similar to those derived using full hierarchical methods.^4,6,29^ Our findings also agree with a recent simulation study evaluating the performance of the bivariate model.^30^ However, our study is more comprehensive including application to real motivating examples, investigation of a broad array of possible models, suggestions for improving model convergence and guidance on how to select an appropriate model. Furthermore, we do not prescribe a limit on the number of studies required to fit a hierarchical model, rather the merit of applying a particular model should be carefully assessed as we have illustrated with our examples.

Our study has some limitations. First, we were not able to fully explore the effect of heterogeneity or varying the threshold. We addressed factors we considered vital, and varied the sample size of studies in a meta-analysis to reflect reality. According to Begg,^31^ the statistical properties of hierarchical models are likely to be most vulnerable when the number of studies is small, and also when sample sizes are highly variable. Second, analyses of the simulated datasets were conducted only in SAS and convergence rates may differ between software packages because of differences in obtaining starting values and model fitting options. Nonetheless, SAS is the software most often used to fit HSROC models in frequentist analyses and we were able to explore several options for improving convergence. Third, when comparing models, we did not limit analyses to datasets that converged across all models. Non-convergence occurred more frequently in challenging datasets where poor model performance (bias, MSE and coverage) can be expected. Therefore, more complex methods with poor convergence rates may be biased or give imprecise estimates. The performance of simpler models with better convergence rates should also be affected but if the models give unbiased and precise estimates, then simpler models are robust and applicable in such situations.

In summary, random effects logistic models should be the default approach for test accuracy meta-analyses. We recommend UREMs for sensitivity and specificity if a bivariate model fails, or a symmetric HSROC model if estimation of a SROC curve is required and the HSROC model fails. If homogeneity can be assumed, the two models can be further simplified to their fixed effect equivalent. However, when prevalence is very low and the number of studies is very small, the results of any meta-analysis should be interpreted with caution.
